# Spectrum of Dyslipidemias in Treatment-Naïve Human Immunodeficiency Virus-Infected Patients Presenting to an HIV Clinic of a Tertiary Care Hospital

**DOI:** 10.7759/cureus.21972

**Published:** 2022-02-07

**Authors:** Sadaf Iqbal, Sadia Salman, Mehwish Akhtar, Amanullah Bhalli, Javeid Iqbal, Ismat Ullah

**Affiliations:** 1 Department of Internal Medicine, Diabetes and Endocrinology, Jinnah Hospital, Allama Iqbal Medical College, Lahore, PAK; 2 Department of Epidemiology and Public Health, Jinnah Hospital, Allama Iqbal Medical College, Lahore, PAK; 3 Department of Medicine, Fatima Memorial Hospital College of Medicine & Dentistry, Lahore, PAK; 4 Department of Family Medicine, Allama Iqbal Medical College, Lahore, PAK

**Keywords:** dyslipidemia, bmi, family history, treatment-naive, hiv infection

## Abstract

Introduction

HIV/AIDS is a major communicable disease worldwide, especially in developing countries where disease prevalence is over 90%. The National AIDS Control Programme of Pakistan reported around 160,000 HIV cases (140,000-190,000) with a 5% prevalence among traditional risks groups. HIV infection is thought to affect lipids metabolism adversely, thus resulting in increased morbidity and mortality. The aim of the study was to find out the frequency and types of dyslipidemia in patients with HIV not taking anti-retroviral therapy, presenting to an HIV clinic at a tertiary care hospital.

Methods

This cross-sectional study was conducted at the HIV clinic of Jinnah Hospital from January 2020 to July 2020. A total of 280 treatment-naïve patients, fulfilling the inclusion protocol, were included through non-probability consecutive sampling after informed consent. Blood samples of 5 mL were taken using aseptic measures and following standard procedure after ensuring overnight fasting by a nurse and were sent immediately to the pathology laboratory of Allama Iqbal Medical College. The results of the lipid profile were collected the next day and noted in the proforma. Dyslipidemia and type of dyslipidemia were recorded as per operational definition. Data were analyzed by SPSS software, version 27.0 (IBM Corp., Armonk, NY). Cross-tabulation was done to assess the relationship of gender, BMI, and family history on dyslipidemia, and a chi-square test was applied to check statistical significance.

Results

Among 280 treatment-naïve HIV-infected patients, the majority of patients were females (52%). The mean duration of HIV was 9.31 + 2.13 months. About 55% of patients had a BMI of more than 25 kg m^2^. A family history of dyslipidemia was found in 62% of the patients. Dyslipidemia was observed in 70% of patients with maximum derangement seen in total cholesterol level (62%). After applying the chi-square test, a significant relation was identified between BMI and family history with dyslipidemia in HIV-infected individuals (p-value = 0.00).

Conclusion

A considerable proportion of treatment-naïve HIV patients have underlying dyslipidemia with a significant relationship with higher BMI and a family history of dyslipidemia. The findings of this study highlight the importance of early screening for dyslipidemia in HIV patients.

## Introduction

Globally, the human immunodeficiency virus (HIV) pandemic has infected 59 million people with 20 million deaths so far. The National AIDS Control Programme reported around 160,000 HIV cases (140,000-190,000) with a prevalence of 0.1%. New HIV-infected people of all ages are reported around 20,000-24,000. The death rate is increasing from 1,400 in 2010 to 6,400 in 2018 [1].

HIV infection causes sickness like mononucleosis with a slew of non-specific symptoms. An estimated 10% to 60% of patients with early HIV infection would have no symptoms [2]. In patients with acute symptomatic HIV infection, the average time between HIV exposure and the start of symptoms is two to four weeks; however, incubation durations of up to 10 months have been observed [3]. A variety of symptoms and signs that are commonly referred to as acute retroviral syndrome may be detected. In a published series, the most common symptoms include headache, fever, rash, sore throat, arthralgia, myalgia, and lymphadenopathy [4-6].

Liu et al. found gradual increasing dyslipidemia in treatment-naïve HIV patients. HIV itself and antiretroviral (ART) therapy can cause dyslipidemia, so extra care should be taken by the clinicians to an early screening of dyslipidemia to improve quality of life [7]. Bekolo et al. discovered in a cross-sectional research of 114 participants that the prevalence of dyslipidemia was 70%, while hypercholesterolemia was found in 34 (30%) individuals [8]. Bourgi et al. observed in a study that patients living with HIV suffer metabolic complications like insulin resistance and increased free fatty acids [9]. Duro et al. concluded that metabolic syndrome is highly likely to occur in HIV-infected patients who are virally repressed than in patients who have an acute infection [10].

Adal et al. concluded in their cross-sectional study that high cholesterol and high triglycerides (TGs) levels were prevalent in ART naïve-infected patients, so lipid levels should be observed regularly in patients whether on or off ART [11]. Souza et al. assessed 190 peer-reviewed scholarly articles. Patients with HIV/AIDS who did not get ART medication had higher levels of total cholesterol, TGs, low-density lipoprotein cholesterol (LDL-c), and high-density lipoprotein cholesterol (HDL-c). Several ART regimens have different effects on lipid metabolism, especially protease inhibitors. As a result, the infection, distinct classes of drugs, and some compounds from the same domain of ART appear to cause differential modifications in lipid metabolism; for example, nevirapine was found to be strongly related with high HDL-c levels, a cardiovascular disease-protective component [12].

As the incidence rate and death rate are increasing in Pakistan because of HIV, according to the latest Joint United Nations Programme on HIV/AIDS (UNAIDS) report, there is a need to address its morbidity and mortality [13]. Lipid disorders are more prevalent among HIV-infected patients whether they are taking ART medications or not. Understanding these dyslipidemias is important to screen or diagnose the cause of lipid disorder, which may lead to life-threatening cardiovascular diseases and will improve the treatment outcomes in HIV-infected patients, thus providing a better quality of life [14].

## Materials and methods

A cross-sectional study was conducted in the HIV clinic at Jinnah Hospital, Lahore from January 2020 to July 2020. The sample size of 280 cases was estimated with a 95% confidence interval, 5% margin of error, and the expected percentage of patients with high total cholesterol levels as 69%.

Treatment-naïve HIV-infected patients for at least six months, aged 20-65 years, were included in the study by non-probability consecutive sampling. Patients with a history of familial dyslipidemia, known dyslipidemia, using lipid-lowering or already on retroviral medication, diabetes, hypertension, or metabolic syndrome were excluded from the study. Informed consent was obtained from them before they were included in the study. The researcher took blood samples using aseptic precautions and regular protocol after assuring eight hours of overnight fasting and promptly submitted them to the pathology laboratory of Allama Iqbal Medical College, Lahore. A pre-designed proforma was used to record the study variables and the demographic information, as well as reports of lipid profiles. The data's confidentiality was ensured. Dyslipidemia and type of dyslipidemia were documented in accordance with the operational criteria.

SPSS software version 27.0 (IBM Corp., Armonk, NY) was used to enter and analyze data. Age was summarized numerically as mean and standard deviation (SD). Qualitative attributes such as gender, dyslipidemia, and its type, i.e., high cholesterol, high TG, low high-density lipoprotein (HDL), high low-density lipoprotein (LDL), and high very-low-density lipoprotein (VLDL) levels, were provided as frequency and percentages. To account for impact modifiers, data were stratified by gender, BMI, and family history of dyslipidemia. The post-stratification chi-square test was used with p-values less than 0.05 as statistically significant.

## Results

Table 1 displays the descriptive statistics of the patient profile of 280 HIV-infected individuals having no ART treatment and aged 25 to 60 years old with a mean of 41.72 ± 10.47 years. The mean height, weight, and BMI were 166.19 + 8.37 cm, 78.06 + 10.32 kg, and 25.15 + 2.84 kg m^2^, respectively. The mean duration of HIV infection was 9.31 + 2.13 months. There were 134 (48%) males and 136 (52%) females. The majority of patients (154, 55%) had a BMI of more than 25 kg m^2^. There were 173 (62%) patients who had a family history of dyslipidemia.

**Table 1:** Descriptive statistics of patient profile (n = 280).

Variables		Mean	Std. deviation
Age		41.72	10.47
Height		166.19	8.37
Weight		78.06	10.32
BMI		25.15	2.84
Duration of HIV		9.31	2.13
Variables		Frequency (n = 280)	Percent (100%)
Gender	Male	134	48%
	Female	146	52%
BMI	BMI > 25	154	55%
	BMI < 25	126	45%
Family history of dyslipidemia	Yes	173	62%
	No	107	38%

Table 2 displays the frequency distribution of dyslipidemia and its spectrum. It was found that 196 individuals (70%) had dyslipidemia, 173 (62%) patients had cholesterol levels greater than 200 mg/dl, 140 (50%) had TG levels greater than 150 mg/dl, 170 (61%) had LDL levels less than 130 mg/dl, and 207 (74%) patients had HDL levels greater than 35 mg/dl.

**Table 2:** Frequency distribution of dyslipidemia and its spectrum. LDL, low-density lipoprotein; HDL, high-density lipoprotein.

Dyslipidemia	Frequency (n = 280)	Percent, 100%
Yes	196	70%
No	84	30%
Cholesterol level		
>200 mg/dl	173	62%
<200 mg/dl	107	38%
Triglyceride level		
>150 mg/dl	140	50%
<150 mg/dl	140	50%
LDL Level		
>130 mg/dl	110	39%
<130 mg/dl	170	61%
HDL Level		
>35 mg/dl	207	74%
<35 mg/dl	73	26%

Table 3 presents the stratification of effect modifiers. There is a significant relationship between BMI, a familial history of dyslipidemia, and dyslipidemia in treatment-naïve HIV-infected patients with p-value = 0.000. On the other hand, gender had no significant relation with dyslipidemia with a p-value of 0.754.

**Table 3:** Stratification of effect modifiers (n = 280).

BMI vs. dyslipidemia				Dyslipidemia		Total	P-value
				Yes	No		
BMI			<25 kg m^2^	55	71	126	
			>25 kg m^2^	141	13	154	p = 0.00
Total				196	84	280	
Family history of dyslipidemia vs. dyslipidemia				Yes	No	Total	
						Family history of dyslipidemia		Yes		149	24	173	
No		47	60	107	p = 0.00		
Total				196	84	280	
Gender vs. dyslipidemia				Yes	No	Total	
Gender	Male			95	39	134	
	Female			101	45	146	p = 0.75
Total				196	84	280	

## Discussion

Our study found an overall higher prevalence (70%) of dyslipidemias in treatment-naïve HIV patients as compared to the general population. Prevalence of dyslipidemias was much more in those HIV treatment-naïve patients who were having a family history of dyslipidemias (p-value = 0.00) or BMI greater than 25 (p-value = 0.00).

Dyslipidemias are a diverse group of lipid metabolism disorders characterized by abnormal levels of various lipid entities in blood. They have gained medical attention due to their pivotal role in the atherosclerosis process. Cholesterol and TGs are the major lipid formulations that are often found deranged in dyslipidemias and form the basis of dyslipidemias classification. Recent literature revealed that the occurrence of opportunistic infections also had an effect on lipid markers and HIV infection is correlated with dyslipidemia, which worsens as the disease develops. In the early stages of HIV infection, HDL-c was found to be lower, with raised TG and atherogenicity index [15].

Teto et al. also said that HIV infection has been shown to have an effect on lipid profile and antioxidant defense. According to the findings, HIV infection is connected to a substantial drop in total antioxidant ability (TAA), LDL-c, HDL-c, and TC. The findings point to increased oxidative stress and lipid peroxidation in HIV-positive Cameroonian patients, as well as a role for circulating recombinant forms (CRFs) in total cholesterol (TC) and malondialdehyde (MDA) levels [16]. Liu et al. found gradually increasing dyslipidemia in treatment-naïve HIV patients, HIV itself, and ART therapy can cause dyslipidemia, so extra care should be taken by the clinicians to an early screening of dyslipidemia to improve quality of life.

Bekolo et al. conducted a cross-sectional study that included 114 HIV-infected people aged 15 years with 83 (73%) being females. Dyslipidemia was found in 70% of people. In our study, the prevalence of dyslipidemias was also 70% but there was no statistically significant gender difference [17]. Calza et al. concluded in a cross-sectional study that in treatment-naïve HIV patients, mostly TGs were raised (44.3%) with low HDL (41%) [18]. Our study showed raised cholesterol (>200 mg/dl) and TGs (>150 mg/dl) levels in 62% and 50% of patients, respectively. According to Muhammad et al., the mean HDL level in the HIV-positive group was substantially lower in the HIV-positive group as compared to the normal population, indicating a higher frequency of dyslipidemia among patients with HIV. In our study, 26.1% of patients were having a serum HDL value <35 mg/dl. Moreover, an LDL level of >130 mg/dl was observed in 39.3% of patients. Serum lipid abnormalities are prominent amongst treatment-naïve HIV-infected individuals in Nigeria [19].

Limitations of the current study are that it is a single-center study done on a limited number of treatment-naïve patients only. Larger multicenter studies are needed to gain further insight into these dyslipidemias and HIV correlation. The findings of this study highlight the importance of early screening for dyslipidemia in HIV patients and their management to reduce cardiovascular complications [20].

## Conclusions

Lipid abnormalities are prevalent in HIV-infected individuals who have not received treatment. Greater BMI and a family history of dyslipidemia were significantly related to the occurrence of dyslipidemia in treatment-naïve HIV-infected patients. So, clinicians need to focus on early diagnosis and screening of dyslipidemia and its management. This will decrease the risk of cardiovascular problems, thus resulting in better survival as per evidence-based management.
